# Phase 1 trial of avelumab (anti-PD-L1) in Japanese patients with advanced solid tumors, including dose expansion in patients with gastric or gastroesophageal junction cancer: the JAVELIN Solid Tumor JPN trial

**DOI:** 10.1007/s10120-018-0903-1

**Published:** 2018-12-04

**Authors:** Toshihiko Doi, Satoru Iwasa, Kei Muro, Taroh Satoh, Shuichi Hironaka, Taito Esaki, Tomohiro Nishina, Hiroki Hara, Nozomu Machida, Yoshito Komatsu, Yasuhiro Shimada, Satoshi Otsu, Shin Shimizu, Morihiro Watanabe

**Affiliations:** 1 National Cancer Center Hospital East, 6-5-1 Kashiwanoha, Kashiwa, Chiba 277-8577 Japan; 2 National Cancer Center Hospital, Chuo-ku, Tokyo, Japan; 3 Aichi Cancer Center Hospital, Chikusa-ku, Nagoya, Japan; 4 Osaka University Hospital, Suita, Osaka Japan; 5 Chiba Cancer Center, Chuo-ku, Chiba, Japan; 6 National Kyushu Cancer Center, Minami-ku, Fukuoka, Japan; 7 Shikoku Cancer Center, Matsuyama, Ehime Japan; 8 Saitama Cancer Center, Kita, Adachi-gun, Saitama, Japan; 9 Shizuoka Cancer Center, Sunto-gun, Shizuoka, Japan; 10 Hokkaido University Hospital, Kita-ku, Sapporo, Japan; 11 Kochi Health Sciences Center, Kochi, Japan; 12 Oita University Hospital, Oita, Japan; 13 Merck Serono Co., Ltd, Tokyo, Japan

**Keywords:** PD-L1, Gastric cancer, Phase 1, Japan, Avelumab

## Abstract

**Background:**

Avelumab is a human anti-PD-L1 IgG1 monoclonal antibody that has shown antitumor activity in several advanced cancers. We report results from JAVELIN Solid Tumor JPN, a phase 1 trial of avelumab in Japanese patients with advanced solid tumors with expansion in patients with advanced gastric cancer/gastroesophageal junction cancer.

**Methods:**

In the dose-escalation part, eligible patients had various previously treated metastatic or advanced solid tumors. In the dose-expansion part, patients had stage IV gastric cancer/gastroesophageal junction adenocarcinoma and disease progression after prior therapy that included a platinum and fluoropyrimidine agent. Patients received avelumab every 2 weeks intravenously at 3, 10, or 20 mg/kg during dose escalation and 10 mg/kg during dose expansion.

**Results:**

Among 17 patients who received avelumab in the dose-escalation part, no dose-limiting toxicities occurred, and the maximum tolerated dose was not reached. 40 patients were enrolled in the dose-expansion part, of whom 21 (52.5%) had received ≥ 3 prior lines of therapy for advanced disease. In these patients, the objective response rate was 10.0% (95% CI, 2.8–23.7%) and median overall survival was 9.1 months (95% CI, 7.2–11.2 months). Three of 40 patients (7.5%) had a grade 3 treatment-related adverse event (alanine aminotransferase increase, anemia, and hyponatremia), and no grade ≥ 4 treatment-related adverse events occurred. Five patients (12.5%) had an immune-related adverse event (all grade 1/2).

**Conclusions:**

Avelumab showed acceptable safety in Japanese patients with advanced solid tumors and clinical activity in patients with advanced gastric cancer/gastroesophageal junction cancer and disease progression after chemotherapy.

**Electronic supplementary material:**

The online version of this article (10.1007/s10120-018-0903-1) contains supplementary material, which is available to authorized users.

## Introduction

Gastric cancer (GC) is the fifth most common cancer and the third leading cause of cancer-related death worldwide [1]. The incidence of GC is higher in Asian populations compared with the rest of the world, as shown by age-standardized incidence rates per 100,000 men and women, respectively, of 35.4 and 13.8 in East Asia [2] and 45.8 and 16.5 in Japan [1] compared with 17.4 and 7.5 worldwide [2]. Because of the high incidence rates of GC, screening is routine in Japan and several other Asian countries, and patients are diagnosed earlier than in non-Asian countries. In population studies of patients with GC/gastroesophageal junction cancer (GEJC), Asian patients appear to survive longer than non-Asian patients, which is likely due in part to earlier diagnosis but may also be due to biological factors [3–6]. GEJC is a cancer distinct from GC but with a similar biology and similar treatments in the advanced setting [7–9]. Recommended treatments for advanced GC/GEJC are generally consistent among countries. First-line therapy usually consists of doublet or triplet chemotherapy including a platinum agent and fluoropyrimidine, with the addition of trastuzumab in patients with human epidermal growth factor receptor 2 (HER2)-positive tumors. Second-line options include taxane, irinotecan, or ramucirumab monotherapy, or paclitaxel plus ramucirumab. Currently, there is no globally accepted standard of care for third-line treatment [9–11]. Japanese guidelines for GC are comparable to those in other regions except for the recommendation of S-1 as fluoropyrimidine of choice for first-line treatment (S-1 remains an investigational agent in North America) and paclitaxel plus ramucirumab as a preferred option for second-line treatment [11, 12]. Triplet regimens are not recommended for general practice in Japan [11]. The overall prognosis for patients with advanced GC/GEJC remains poor, particularly in countries without early screening programs [10, 11, 13], and treatment advances in recent years have been limited.

The immune checkpoint proteins programmed cell death 1 ligand 1 (PD-L1) and programmed cell death 1 protein (PD-1) appear to play significant roles in GC/GEJC pathobiology, with PD-L1 being expressed in 30–45% of GC/GEJC tumors [14, 15]. In some studies, PD-L1 expression has been associated with a worse prognosis in GC/GEJC, including an increased incidence of disease progression and shorter survival [16–18], although other studies have reported conflicting findings [14, 15]. In addition, PD-1 is upregulated on T cells in patients with GC [19], further suggesting that blockade of the PD-L1 and PD-1 interactions is a rational therapeutic strategy.

Antibodies targeting PD-L1 or PD-1, which can reactivate suppressed antitumor immune responses, have become an established part of treatment for various cancers, and these agents have also shown clinical activity in patients with GC/GEJC. In a recent phase 3 trial in 493 patients with advanced GC performed in Japan, South Korea, and Taiwan (ATTRACTION-2), nivolumab (anti-PD-1) administered as third- or later-line treatment was associated with longer overall survival (OS) than placebo (median, 5.26 vs 4.14 months; *P* < 0.0001); objective response rates (ORRs) for nivolumab vs placebo were 11.2% vs 0%, respectively [20]. Based on this study, nivolumab was approved in Japan, South Korea, and Taiwan for the treatment of patients with unresectable advanced or recurrent GC and disease progression after chemotherapy. In a cohort of the phase 2 KEYNOTE-059 trial, pembrolizumab was administered as third- or later-line treatment in patients with GC/GEJC (*n* = 259). The ORR was 11.6% overall, and was 15.5% and 6.4% in patients with PD-L1+ and PD-L1− tumors, respectively [21]. Based on these results, pembrolizumab was approved by the US Food and Drug Administration for the treatment of patients with PD-L1+ GC/GEJC and disease progression after ≥ 2 prior lines of therapy. However, the global phase 3 trial, KEYNOTE-061, did not demonstrate superior OS for second-line pembrolizumab compared with paclitaxel in patients with GC/GEJC (median, 9.1 vs 8.3 months; *P* < 0.0421) [22].

Avelumab is a human IgG1 monoclonal antibody with a wild-type Fc region that blocks the PD-L1/PD-1 interaction [23]. In addition to reactivating adaptive immune responses by inhibiting this interaction, preclinical models show that avelumab can also induce innate effector cell functions, a characteristic not seen with other approved anti-PD-L1/PD-1 antibodies; thus, avelumab may engage both the adaptive and innate immune systems [24, 25]. Avelumab has been approved in various countries for the treatment of metastatic Merkel cell carcinoma and locally advanced or metastatic urothelial carcinoma with progression following platinum-containing chemotherapy [26–28].

International studies of avelumab in patients with advanced GC/GEJC have been reported. In a cohort from a large phase 1 trial, avelumab showed antitumor activity and an acceptable safety profile when administered as second-line or switch-maintenance treatment to patients with or without disease progression after first-line chemotherapy [29]. In a recently reported phase 3 trial, JAVELIN Gastric 300, avelumab administered as third-line treatment for GC/GEJC did not show superior efficacy compared with physician’s choice of chemotherapy (median OS, 4.6 vs 5.0 months; *P* < 0.81) [30].

Here, we report data from a trial of avelumab in Japanese patients (JAVELIN Solid Tumor JPN), including dose escalation in patients with various advanced solid tumors and dose expansion in a cohort of patients with GC/GEJC.

## Methods

### Study design and patients

JAVELIN Solid Tumor JPN is a phase 1, open-label, multicenter trial performed in Japan (NCT01943461). In the dose-escalation part, eligible patients had histologically or cytologically confirmed metastatic or locally advanced solid tumors for which no standard therapy existed or standard therapy had failed. Initial patients in the dose-expansion part had histologically or cytologically confirmed, unresectable, measurable, stage IV GC/GEJC adenocarcinoma and disease progression after 1 or 2 prior chemotherapy regimens that included both platinum and fluoropyrimidine therapy. Following a protocol amendment, eligible patients had disease progression after both first- and second-line treatment that included platinum and fluoropyrimidine treatment followed by taxane or irinotecan treatment. In the dose-expansion part, patients with severe peritoneal metastases (defined as clinical ileus or subileus or moderate–severe ascites) were ineligible, and patients were required to have fresh or archival tumor samples (formalin-fixed, paraffin-embedded tissue) available. Other inclusion criteria in both parts of the trial included age ≥ 20 years, Eastern Cooperative Oncology Group (ECOG) performance status of 0 or 1, estimated life expectancy ≥ 3 months, and adequate hematologic, hepatic, and renal function. Exclusion criteria included central nervous system metastases, prior therapy with any antibody or drug targeting a T-cell coregulatory protein (immune checkpoint; eg, PD-L1/PD-1), or other anticancer therapy ≤ 30 days before start of study treatment (14 days in the dose-expansion part). Any use of steroids was tapered before study treatment except in patients with adrenal insufficiency, who could continue treatment at a physiological replacement dose.

### Procedures and assessments

The dose-escalation part was performed using a standard 3 + 3 design with avelumab doses of 3, 10, and 20 mg/kg administered by 1-h intravenous infusion every 2 weeks (Q2W). After analysis of tolerability and pharmacokinetics (PK), the dose-expansion part started enrollment and all patients received avelumab 10 mg/kg Q2W. All patients (dose-escalation and dose-expansion cohorts) continued to receive their assigned dose throughout the trial. Premedication with diphenhydramine 25–50 mg and acetaminophen 650 mg (modified based on local treatment standards and guidelines) was required 30–60 min before all infusions of avelumab. In the dose-escalation part, the first patient treated at each dose level was observed for 16 days (until 48 h after the second dose) for the occurrence of any dose-limiting toxicity (DLT) before the second patient began treatment. The second and third patients were treated ≥ 48 h apart. Adverse events (AEs) and laboratory abnormalities were classified and graded according to the National Cancer Institute Common Terminology Criteria for Adverse Events version 4.0. A DLT was defined as any grade ≥ 3 AE that occurred during the first 3 weeks of treatment in the dose-escalation part (ie, the DLT observation period) and was considered related to avelumab treatment by the investigator or the sponsor. The maximum tolerated dose (MTD) was defined as the highest dose level at which ≤ 1 of 6 evaluable patients experienced a DLT.

Patients were treated until progression or unacceptable toxicity or until other protocol-specified criteria for withdrawal were met. Treatment was discontinued for any grade 4 AE, except for single laboratory values out of the normal range that were deemed unrelated to study treatment, without clinical correlate, and that resolved in ≤ 7 days with medical management. Treatment was also discontinued for any grade 3 AE except for (1) transient (≤ 6 h) influenza-like symptoms or pyrexia controlled with medical management; (2) fatigue, local infusion-related reaction (IRR), headache, nausea, or emesis that resolved to grade ≤ 1 in ≤ 24 h; (3) single laboratory values out of the normal range that were deemed unrelated to study treatment and without clinical correlate (excluding a grade ≥ 3 increase in liver enzyme concentrations) that resolved to grade ≤ 1 in ≤ 7 days after medical management has been initiated; (4) tumor flare phenomena (local pain, irritation, or localized rash at a known or suspected tumor site); or (5) worsening of ECOG performance score to ≥ 3 that did not resolve to ≤ 2 by the last day of the following treatment cycle. Grade 2 AEs were managed by treatment delays; events that did not resolve to grade ≤ 1 by the end of the following treatment cycle or that recurred led to permanent discontinuation of avelumab (except for hormone insufficiencies that could be managed by replacement therapy).

Safety assessments included documentation of AEs, physical examination, clinical laboratory tests (hematology, hepatic panels, and serum chemistry), and documentation of concurrent medications. A serious AE (SAE) was defined as any untoward event that was life-threatening, required hospitalization, resulted in disability, was a congenital anomaly, resulted in death, or was otherwise considered as medically important. Immune-related AEs (irAEs) were identified using a prespecified list of Medical Dictionary for Regulatory Activities (MedDRA) terms followed by a comprehensive medical review. IRRs were analyzed using an expanded definition that included both a prespecified list of MedDRA preferred terms (reactions occurring post-infusion on the same day or following day) and related signs and symptoms (based on specified MedDRA terms) that occurred on the day of infusion and resolved ≤ 2 days.

Clinical activity was assessed by investigators using Response Evaluation Criteria in Solid Tumors (RECIST) version 1.1 and modified immune-related response criteria to determine the best overall response and progression-free survival (PFS) duration. Radiographic tumor assessments were performed at baseline and then every 6 weeks. For patients who had a partial response (PR) or complete response (CR), a confirmatory CT or MRI scan was done no sooner than 28 days after assessment and preferably at the scheduled 6-week interval. Blood samples for analysis of avelumab concentrations in serum were drawn 6 h before and at the end of infusion (peak value) during weeks 1, 3, 5, 7, and 13, and then every 6 weeks. In the dose-escalation part, additional samples were taken 0.5, 1, 2, 4, 6, 12, 24, 36, 48, and 168 h after the first infusion.

In patients enrolled in the dose-expansion part, PD-L1 expression was assessed in fresh and archival tumor samples using a proprietary immunohistochemistry assay (Dako PD-L1 immunohistochemistry 73-10 pharmDx; Carpinteria, CA) based on an anti-PD-L1 rabbit monoclonal antibody clone (73-10) under license to Merck KGaA [31]. In this report, PD-L1 status was defined using a cutoff of ≥ 1% of tumor cells showing partial or complete membrane PD-L1 staining of any intensity.

### Outcomes

In the dose-escalation part, the primary endpoint was occurrence of DLTs during the first 3 weeks of treatment. Secondary endpoints included safety and tolerability, best overall response per investigator assessment (defined as best response obtained among all tumor assessments after the start of treatment with avelumab until documented disease progression), immune-related best overall response (using modified immune-related response criteria derived from RECIST v1.1) [32, 33], PFS duration, OS, PD-L1 expression, immunogenicity, and PK profile.

### Statistical methods

Planned enrollment in this study was ≤ 18 patients in the dose-escalation part (per 3 + 3 design) and ≤ 40 patients in the dose-expansion part. For the dose-expansion part, the sample size was selected to provide a probability of ≥ 87% to observe any AEs occurring in ≥ 5% of patients, and to provide 95% Clopper–Pearson CIs for potential ORRs (defined as the proportion of patients with a PR or CR), eg, 10% (95% CI, 2.8–23.7%) or 15% (95% CI, 5.7–29.8%). Safety and activity were analyzed in all patients who received ≥ 1 dose of avelumab. In the dose-escalation part, the DLT analysis set was defined as all patients whose data were used to implement the dose-escalation schedule; patients were required to have received all trial treatment administrations in the DLT observation period or to have stopped treatment because of a DLT in the DLT observation period. Change in the sum of target lesion diameters from baseline over time was evaluated in patients with a baseline tumor assessment and ≥ 1 postbaseline assessment. Time-to-event endpoints were estimated with the Kaplan–Meier method, and CIs for the median were calculated using the Brookmeyer–Crowley method. *P* values for the association between categorical variables were determined using Fisher’s exact test. PK parameters were estimated using WinNonlin^®^ (Certera; Princeton, NJ, USA) version 5.0 or higher.

## Results

### Patients

In the dose-escalation and dose-expansion cohorts, the data cutoff date was August 10, 2016. In the dose-escalation part, 17 patients with various advanced solid tumors received avelumab Q2W at doses of 3 mg/kg (*n* = 5), 10 mg/kg (*n* = 6), or 20 mg/kg (*n* = 6) (Table 1). Two of the first three patients assigned to the 3 mg/kg cohort were subsequently found to have received steroids and were, therefore, not evaluable for DLT assessment; thus, two additional patients were treated at this dose level to achieve a cohort of three DLT-evaluable patients. Six patients were treated at 10 and 20 mg/kg to further investigate the safety of avelumab. All 17 patients had received prior therapy; 13 (76.5%) and 6 (35.3%) had received ≥ 3 or ≥ 4 prior lines for advanced disease, respectively. Median duration of avelumab treatment for the 3, 10, and 20 mg/kg dose levels was 3.9, 12.2, and 2.8 months, respectively. Three patients (1 from each dose level) were still receiving treatment at data cutoff. The most common reason for treatment discontinuation was disease progression [3 mg/kg, *n* = 3 (60.0%); 10 mg, *n* = 5 (83.3%); and 20 mg/kg, *n* = 4 (66.7%)]; other reasons were death [3 mg/kg, *n* = 1 (20.0%)] and patient decision to receive a different treatment [20 mg/kg, *n* = 1 (16.7%)].

**Table 1:** Baseline characteristics

Characteristics	Dose escalation (*n* = 17)			Dose expansion (*N* = 40)
	3 mg/kg (*n* = 5)	10 mg/kg (*n* = 6)	20 mg/kg (*n* = 6)	
Median age (range), years	46 (32–69)	62 (30–67)	67 (56–74)	63 (37–77)
< 65 years, *n* (%)	3 (60.0)	5 (83.3)	2 (33.3)	22 (55.0)
≥ 65 years, *n* (%)	2 (40.0)	1 (16.7)	4 (66.7)	18 (45.0)
Sex, *n* (%)				
Male	3 (60.0)	4 (66.7)	3 (50.0)	29 (72.5)
Female	2 (40.0)	2 (33.3)	3 (50.0)	11 (27.5)
ECOG performance status, *n* (%)				
0	5 (100.0)	4 (66.7)	5 (83.3)	23 (57.5)
1	0	2 (33.3)	1 (16.7)	17 (42.5)
Median time since first diagnosis (range), years	2.9 (1.9–6.1)	4.5 (1.3–7.7)	2.9 (1.3–22.3)	1.5 (0.5–9.1)
Median time since diagnosis of metastatic disease (range), years	1.5 (0.3–6.1)	3.1 (0.8–7.7)	2.9 (1.3–14.6)	1.4 (0.1–8.4)
Site of primary tumor, *n* (%)				
Breast	1 (20.0)	0	0	0
Choroid	0	0	1 (16.7)	0
Colon	1 (20.0)	0	0	0
Esophagus	0	1 (16.7)	0	0
Gastroesophageal junction	0	0	0	5 (12.5)
Lung	0	2 (33.3)	1 (16.7)	0
Nasal cavity	1 (20.0)	0	0	0
Rectum	0	1 (16.7)	0	0
Skin	2 (40.0)	0	0	0
Small intestine	0	0	1 (16.7)	0
Stomach	0	2 (33.3)	3 (50.0)	35 (87.5)
Number of prior anticancer therapy lines for metastatic or locally advanced disease, *n* (%)				
1	1 (20.0)	0	0	3 (7.5)
2	0	1 (16.7)	1 (16.7)	14 (35.0)
3	2 (40.0)	3 (50.0)	2 (33.3)	14 (35.0)
≥ 4	1 (20.0)	2 (33.3)	3 (50.0)	7 (17.5)
Missing	1 (20.0)	0	0	2 (5.0)
PD-L1 expression (≥ 1% of tumor cells), *n* (%)	Not assessed	Not assessed	Not assessed	
Negative				27 (67.5)
Positive				11 (27.5)
Not evaluable				2 (5.0)

*ECOG* Eastern Cooperative Oncology Group

After analysis of safety and PK data, patients were enrolled in the dose-expansion part. Overall, 40 patients with advanced GC/GEJC, who had a median age of 63 years (range, 37–77 years), were treated with avelumab 10 mg/kg Q2W (Table 1). Most patients [*n* = 35 (87.5%)] had GC and the remainder [*n* = 5 (12.5%)] had GEJC. HER2 status was positive in 11 patients (27.5%), negative in 20 patients (50.0%), and equivocal or not available in 9 patients (22.5%). All 40 patients had received prior therapy, including ≥ 3 or ≥ 4 prior lines for advanced disease in 21 patients (52.5%) and 7 patients (17.5%), respectively. Median duration of avelumab treatment was 2.7 months (range, 0.5–21.4 months) and median follow-up was 19.3 months (range, 0.4–22.9 months). Two patients (5.0%) were still receiving treatment at data cutoff. The most common reason for treatment discontinuation was disease progression [*n* = 33 (82.5%)]; other reasons were AE [*n* = 4 (10.0%)] and death [*n* = 1 (2.5%)].

### Safety: dose-escalation cohort

Of 15 patients in the dose-escalation cohort enrolled in the DLT analysis set (3 mg/kg, *n* = 3; 10 mg/kg, *n* = 6; 20 mg/kg, *n* = 6), no patient had a DLT and the MTD was not reached. Of 17 patients in the full dose-escalation cohort, 16 patients (94.1%) had an AE of any grade, of whom 11 patients (64.7%) had a treatment-related AE (TRAE) of any grade: 3 patients (60.0%) at 3 mg/kg, 5 patients (83.3%) at 10 mg/kg, and 3 patients (50.0%) at 20 mg/kg (Table 2). No patient in the dose-escalation part had a grade ≥ 3 TRAE.

**Table 2:** Treatment-related adverse events (TRAEs) occurring at any grade in ≥ 10% of patients at any dose level and infusion-related reactions in the dose-escalation cohort (*N* = 17)

	3 mg/kg (*n* = 5)	10 mg/kg (*n* = 6)	20 mg/kg (*n* = 6)	Overall (*N* = 17)
Any TRAE, *n* (%)^a^	3 (60.0)	5 (83.3)	3 (50.0)	11 (64.7)
Rash maculopapular	2 (40.0)	1 (16.7)	1 (16.7)	4 (23.5)
Stomatitis	1 (20.0)	3 (50.0)	0	4 (23.5)
WBC count decreased	1 (20.0)	2 (33.3)	0	3 (17.6)
Anemia	1 (20.0)	0	1 (16.7)	2 (11.8)
Dermatitis acneiform	1 (20.0)	1 (16.7)	0	2 (11.8)
Headache	0	1 (16.7)	1 (16.7)	2 (11.8)
Pyrexia	0	1 (16.7)	1 (16.7)	2 (11.8)
Infusion-related reaction, *n* (%)^b^	1 (20.0)	2 (33.3)	2 (33.3)	5 (29.4)

No grade ≥ 3 TRAEs occurred

*WBC* white blood cell

^a^The incidence of treatment-related infusion-related reaction based on the single MedDRA preferred term is not listed.

^b^Composite term; includes AEs categorized as infusion-related reaction, drug hypersensitivity, or hypersensitivity reaction that occurred on the day of infusion or day after infusion, in addition to signs and symptoms of infusion-related reaction that occurred on the same day of infusion and resolved within 2 days (including AEs classified by investigators as related or unrelated to treatment)

Across all dose-escalation levels, IRRs identified via an expanded definition occurred in 5 patients (29.4%; 3 mg/kg, *n* = 1; 10 mg/kg, *n* = 2; 20 mg/kg, *n* = 2), all of which were grade 1 or 2 and occurred in patients in the DLT analysis set. Other TRAEs occurring in ≥ 20% of patients were rash maculopapular [*n* = 4 (23.5%)] and stomatitis [*n* = 4 (23.5%)]. Two patients (11.8%) had an irAE: 1 patient (3 mg/kg) had grade 2 rash maculopapular, and 1 patient (20 mg/kg) had grade 3 aspartate aminotransferase increase and grade 1 alanine aminotransferase (ALT) increase. Two patients (11.8%) had serious AEs, which were not treatment-related, and no patient had an AE that led to death.

### Safety: dose-expansion cohort

All 40 patients in the dose-expansion cohort had an AE of any grade, of which 32 patients (80.0%) had a TRAE of any grade (Table 3). IRRs identified via an expanded definition occurred in 12 patients (30.0%); all were grade 1 or 2, occurred at the first (*n* = 11) or second (*n* = 1) infusion, and did not lead to treatment discontinuation. Other TRAEs occurring at any grade in ≥ 10% of patients were pruritus [*n* = 6 (15.0%)], pyrexia [*n* = 5 (12.5%)], and rash [*n* = 4 (10.0%)]. Three patients (7.5%) had a grade 3 TRAE (ALT increase, anemia, and hyponatremia); no grade ≥ 4 TRAEs occurred. Five patients (12.5%) had an irAE, all of which were grade 1 or 2 (Table 3). Pruritus (*n* = 3) and maculopapular rash (*n* = 2) were the only irAEs that occurred in > 1 patient. Ten patients (25.0%) had a serious AE, and three patients (7.5%) had an AE that led to death [myocardial infarction, multiple organ failure, and aggravation of underlying disease (GC)]; none of these were treatment-related.

**Table 3:** Treatment-related adverse events (TRAEs; any grade in ≥ 10% of patients or grade ≥ 3 in any patient), infusion-related reactions, and immune-related adverse events (AEs; any grade in any patient) in patients with GC/GEJC in the dose-expansion cohort (*N* = 40)

*N* = 40	Any grade	Grade 3
Any TRAE, n (%)^a^	32 (80.0)	3 (7.5)
Pruritus	6 (15.0)	0
Pyrexia	5 (12.5)	0
Rash	4 (10.0)	0
Anemia	2 (5.0)	1 (2.5)
Alanine aminotransferase increased	1 (2.5)	1 (2.5)
Hyponatraemia	1 (2.5)	1 (2.5)
Infusion-related reaction^b^	12 (30.0)	0
Any immune-related AE, *n* (%)	5 (12.5)	0
Pruritus	3 (7.5)	0
Rash maculopapular	2 (5.0)	0
Rash	1 (2.5)	0
Hyperthyroidism	1 (2.5)	0
Hypothyroidism	1 (2.5)	0
Secondary adrenocortical insufficiency	1 (2.5)	0

No grade ≥ 4 TRAEs occurred

^a^The incidence of treatment-related infusion-related reaction based on the single MedDRA preferred term is not listed

^b^Composite term; includes AEs categorized as infusion-related reaction, drug hypersensitivity, or hypersensitivity reaction that occurred on the day of infusion or day after infusion, in addition to signs and symptoms of infusion-related reaction that occurred on the same day as the infusion and resolved within 2 days (including AEs classified by investigators as related or unrelated to treatment)

### Pharmacokinetics analyses

All 57 patients were evaluable for PK analysis. In the dose-escalation cohort (Online Resource 1), avelumab exposure in terms of maximum serum concentration observed postdose (*C*_max_) and area under the concentration–time curve from time 0–2 weeks (AUC_0–336 h_) after first administration increased in an approximately dose-proportional fashion between 3 and 20 mg/kg doses, and the estimated geometric mean apparent half-life (*t*_1/2_) ranged from 94 to 122 h (corresponding to 3.9–5.1 days). Mild drug accumulation following multiple infusions was observed (shown by an increase in mean *C*_trough_ over time; Online Resource 2), consistent with the estimated *t*_1/2_ from first administration and the Q2W dosing regimen. Median trough concentration levels (*C*_trough_) of avelumab showed a high degree of interpatient variability (coefficient of variation shown in Online Resource 1). Following administration of avelumab at 10.0 mg/kg, median *C*_trough_ appeared to be higher in the dose-escalation part (range, 20.6–54.7 µg/mL) than in the dose-expansion part (range, 14.12–22.13 µg/mL).

### Antitumor activity

In the dose-escalation cohort, 3 patients (17.6%) had a confirmed objective response (all PRs), comprising single patients with melanoma (3 mg/kg), esophageal squamous cell carcinoma (10 mg/kg), and GC (20 mg/kg). 11 additional patients had stable disease (SD) as best overall response.

**Fig. 1:**
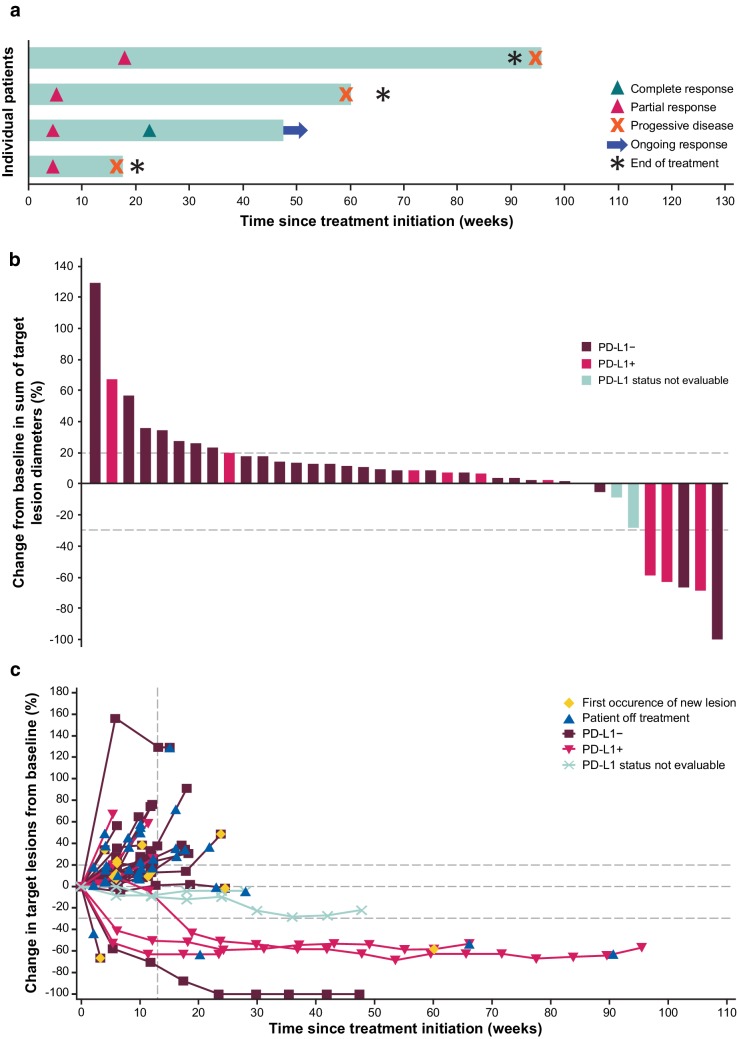
Antitumor activity of avelumab in patients with GC/GEJC in the dose-expansion cohort (*N* = 40). **a** Time to and duration of response in responding patients (*n* = 4). **b** Best change in target lesions from baseline by PD-L1 status (≥ 1% cutoff; *n* = 38 evaluable). **c** Change in target lesions from baseline over time by PD-L1 status (≥ 1% cutoff; *n* = 38 evaluable)

In the dose-expansion cohort, the ORR was 10.0% (95% CI, 2.8–23.7%), including CR in 1 patient (2.5%) and PR in 3 patients (7.5%) (Table 4). Of the 4 responding patients, 3 had received 2 prior lines of systemic therapy for advanced disease, and 1 had received 4 prior lines. The ORR in patients with 1, 2, or ≥ 3 prior lines was 0% (0 of 3; 95% CI, 0.0–70.8%), 21.4% (3 of 14; 95% CI, 4.7–50.8%), and 4.8% (1 of 21; 95% CI, 0.1–23.8%), respectively. Of responding patients, 1 had a HER2+ tumor and 3 had HER2− tumors. Duration of response in the expansion cohort ranged from 2.8 to 17.7 months, with 1 response ongoing at data cutoff (Fig. 1a). An additional 17 patients (42.5%) had a confirmed best overall response of SD, and the disease control rate was 52.5% (Table 4). The immune-related ORR was 10.0% (95% CI, 2.8–23.7%), and 21 (52.5%) additional patients had a best overall response of immune-related SD. A ≥ 30% tumor reduction occurred in 5 (13.2%) of 38 evaluable patients (Fig. 1b, c). ORRs in patients with PD-L1+ or PD-L1− tumors were 27.3% (3 of 11; 95% CI, 6.0–61.0%) vs 3.7% (1 of 27; 95% CI, 0.1–19.0%; *P* = .065), respectively.

**Table 4:** Confirmed objective responses in patients with GC/GEJC in the dose-expansion cohort (*N* = 40)

Response	*N* = 40
Best overall response, *n* (%)	
Complete response	1 (2.5)
Partial response	3 (7.5)
Stable disease	17 (42.5)
Progressive disease	17 (42.5)
Not evaluable	2 (5.0)
ORR, % (95% CI)	10.0 (2.8–23.7)
Disease control rate, %	52.5
Immune-related best overall response, *n* (%)	
Complete response	1 (2.5)
Partial response	3 (7.5)
Stable disease	21 (52.5)
Progressive disease	9 (22.5)
Not evaluable	6 (15.0)
Immune-related ORR, % (95% CI)	10.0 (2.8–23.7)

*ORR* objective response rate

**Fig. 2:**
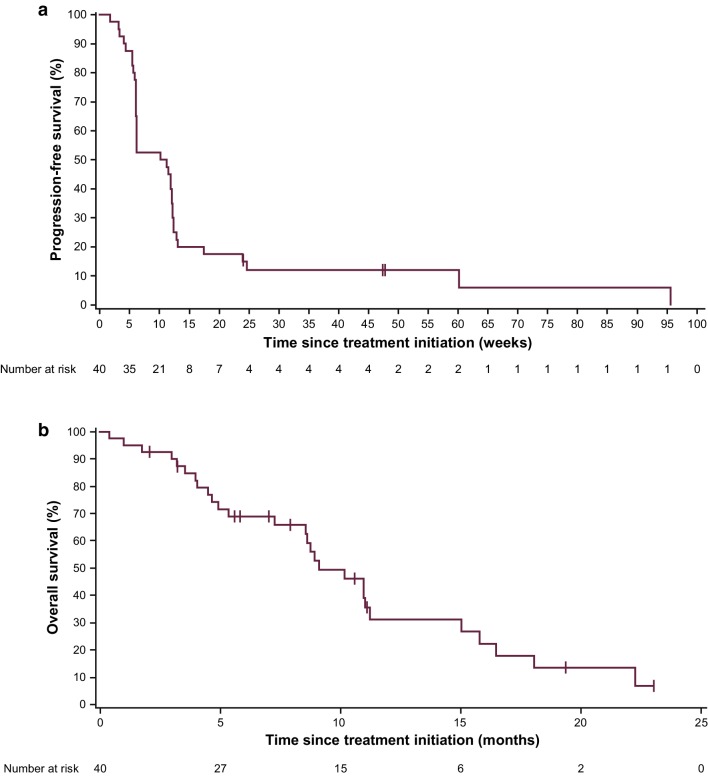
Kaplan–Meier estimates of **a** progression-free survival and **b** overall survival in patients with GC/GEJC in the dose-expansion cohort (*n* = 40)

In the dose-expansion cohort, median PFS was 2.4 months (95% CI, 1.4–2.8 months), and the 3-month PFS rate was 35.0% (95% CI, 20.8–49.6%) (Fig. 2a). Median PFS in patients with PD-L1+ or PD-L1− tumors was 1.4 months (95% CI, 0.7–4.0 months) and 2.6 months (95% CI, 1.4–2.8 months), respectively (Online Resource 3a). Median OS in all patients (*n* = 40) was 9.1 months (95% CI, 7.2–11.2 months), and the 12-month OS rate was 31.0% (95% CI, 15.6–47.8%) (Fig. 2b). Median OS in patients with PD-L1+ or PD-L1− tumors was 10.9 months (95% CI, 1.0 months–not estimable) and 9.1 months (95% CI, 4.9–11.0 months), respectively (Online Resource 3b).

## Discussion

In this single-arm study in Japanese patients, avelumab had an acceptable safety profile at doses up to 20 mg/kg Q2W. No DLTs were reported, and an MTD was not reached. The rate of grade 3 TRAEs was low (0% in the dose-escalation part and 7.5% in the dose-expansion part), and no grade ≥ 4 TRAEs were reported.

PK parameters, including *C*_trough_, *C*_max_, and end-of-infusion levels, were lower than seen in previous studies with the same dose levels in non-Asian patients, likely due to the lower mean body weight and associated higher relative blood volume in Japanese patients [34]. However, the safety profile of avelumab in Japanese patients in this study was consistent with that of previously reported studies in global populations [23, 32]. The relatively lower C_trough_ in the dose-expansion cohort, compared with that in the dose-escalation cohort at the same dose level, may have been due to different disease characteristics in patients with GC/GEJC vs other tumor types, or it may have been a sampling artifact due to the small number of patients (*n* = 6) who received the 10 mg/kg dose in the dose-escalation part.

Based on safety and PK findings from this study, and with consideration of PK and target occupancy data from an international phase 1 study of avelumab [23], the 10 mg/kg Q2W dose was selected for further study in a cohort of Japanese patients with advanced GC/GEJC. In these heavily pretreated patients, avelumab showed durable antitumor activity, including an ORR of 10.0% (including responses in patients with HER2+ and HER2− tumors), median PFS of 2.4 months, and median OS of 9.1 months (12-month OS rate of 31%). The results of this trial are consistent with findings from a global cohort of patients with GC/GEJC treated in the JAVELIN Solid Tumor trial [29], and previous phase 1 and 2 studies of anti-PD-1 antibodies [21, 35, 36]. In our study, ORR was numerically higher in patients with PD-L1+ tumors, although responses were also seen in patients with PD-L1− tumors. It should be noted that PD-L1 expression status in this study was assessed using an assay (73-10) that is distinct from those used in trials of other anti-PD-1 or PD-L1 agents. Moreover, PD-L1 status in this study was determined based on tumor cell expression only, whereas in the KEYNOTE-059 study of pembrolizumab, PD-L1 status was based on expression on tumor cells and immune cells (ie, combined proportion score) [21], hampering any cross-trial comparison of efficacy trends in GC/GEJC based on PD-L1 status.

As discussed earlier, 2 global phase 3 trials in patients with previously treated advanced GC/GEJC (KEYNOTE-061 and JAVELIN Gastric 300) that compared anti-PD-1/PD-L1 antibody treatment (pembrolizumab or avelumab) with standard second-line or third-line chemotherapy did not meet their primary endpoints [22, 30]. However, several phase 3 studies are ongoing to investigate alternative uses of checkpoint inhibitors in the treatment of GC/GEJC. For example, an ongoing phase 3 trial (JAVELIN Gastric 100) is assessing switch-maintenance treatment with avelumab vs continuation of first-line chemotherapy. Other ongoing phase 3 studies in patients with advanced GC/GEJC include a trial of second-line pembrolizumab monotherapy vs paclitaxel in Asian patients (KEYNOTE-063), a trial of pembrolizumab as first-line treatment in combination with chemotherapy (KEYNOTE-062), a trial of nivolumab in combination with chemotherapy as first-line treatment in Asian patients (ATTRACTION-04), and a trial of first-line nivolumab plus ipilimumab or chemotherapy vs chemotherapy alone (CheckMate-649). Results from ongoing studies will help to define an appropriate role for checkpoint inhibitors in the treatment of GC/GEJC.

## Electronic supplementary material

Below is the link to the electronic supplementary material.

Supplementary material 1 (DOCX 222 KB)
