# Supplementary material for: Phase 1 trial of avelumab (anti-PD-L1) in Japanese patients with advanced solid tumors, including dose expansion in patients with gastric or gastroesophageal junction cancer: the JAVELIN Solid Tumor JPN trial
Source: Gastric Cancer. 2018 Dec 4;22(4):817–27. doi: 10.1007/s10120-018-0903-1 (PMC6570778; doi:10.1007/s10120-018-0903-1)

**Supplementary information**

**Online Resource 1** PK parameters after the first dose of avelumab in the dose-escalation cohort with ≥169-hour sampling

| **Variable** | **Dose cohort (mg/kg)** | **Mean** | **Median** | **SD** | **n** | **GM** | **CV% GM** |
| --- | --- | --- | --- | --- | --- | --- | --- |
| C_max_, μg/mL | 3  10  20 | 65.2  182  462 | 70.5  177  471 | 13.7  35.4  60.9 | 5  6  6 | 64  179  459 | 22.2  19.6  13.6 |
| C_trough_, μg/mL | 3  10  20 | 3.32  23.9  44.4 | 3.54  20.6  43.2 | 2.4  15.5  15.0 | 4  6  6 | 2.49  18.9  42.4 | 121.6  99.2  34.8 |
| AUC_0-t_ (h*μg/mL) | 3  10  20 | 5320  21400  47800 | 5520  18500  44700 | 1610  8510  11500 | 5  6  6 | 5120  20100  46800 | 32.5  40.0  23.1 |
| AUC_0-336hr_ (h*μg/mL) | 3  10  20 | 5620  21400  48000 | 5470  18500  45200 | 1340  8500  11400 | 5  6  6 | 5480  20100  47000 | 25.5  40.0  22.8 |
| AUC_0-∞_ (h*μg/mL) | 3  10  20 | 6220  26100  55100 | 6000  21500  52300 | 1570  11800  13900 | 5  6  6 | 6040  24000  53700 | 27.5  47.4  24.3 |
| t_1/2_, hours | 3  10  20 | 97.6  126  113 | 104  141  118 | 29.1  33.9  12.4 | 5  6  6 | 94  122  112 | 31.7  33.1  11.6 |

*AUC_0-t_* area under the concentration-time curve from time zero to the last quantifiable concentration, *AUC_0-336hr_* area under the concentration-time curve from time zero to 2 weeks, *AUC_0-∞_* area under the concentration-time curve from time zero extrapolated to infinity, *C_max_* maximum serum concentration observed postdose, *C_trough_* trough concentration levels, *CV* coefficient of variation, *GM* geometric mean, *PK* pharmacokinetics, *SD* standard deviation, *t_1/2_* half-life

**Online Resource 2** C_trough_ of avelumab following IV infusion Q2W, grouped by cohort


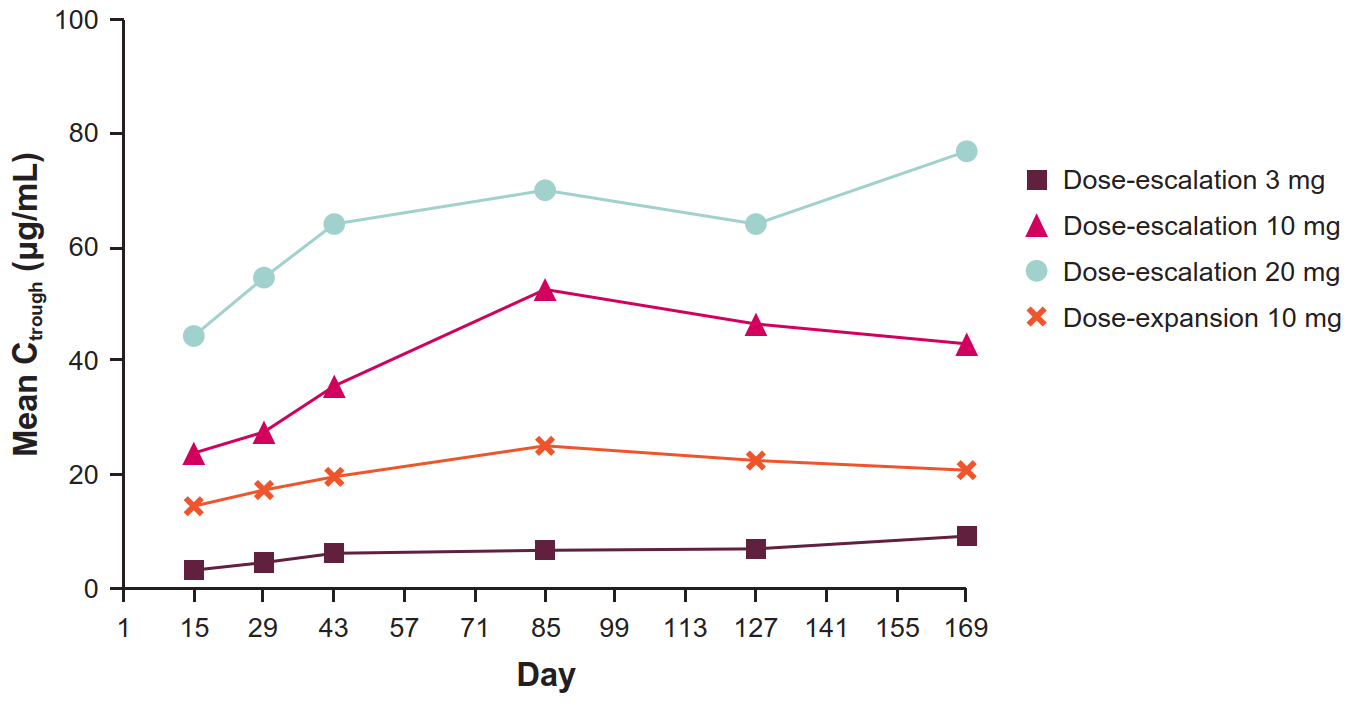


**Online Resource 3** Kaplan–Meier estimates of (a) progression-free survival and (b) overall survival based on a ≥1% cutoff in patients with GC/GEJC in the dose-expansion cohort (n=40)

**a**
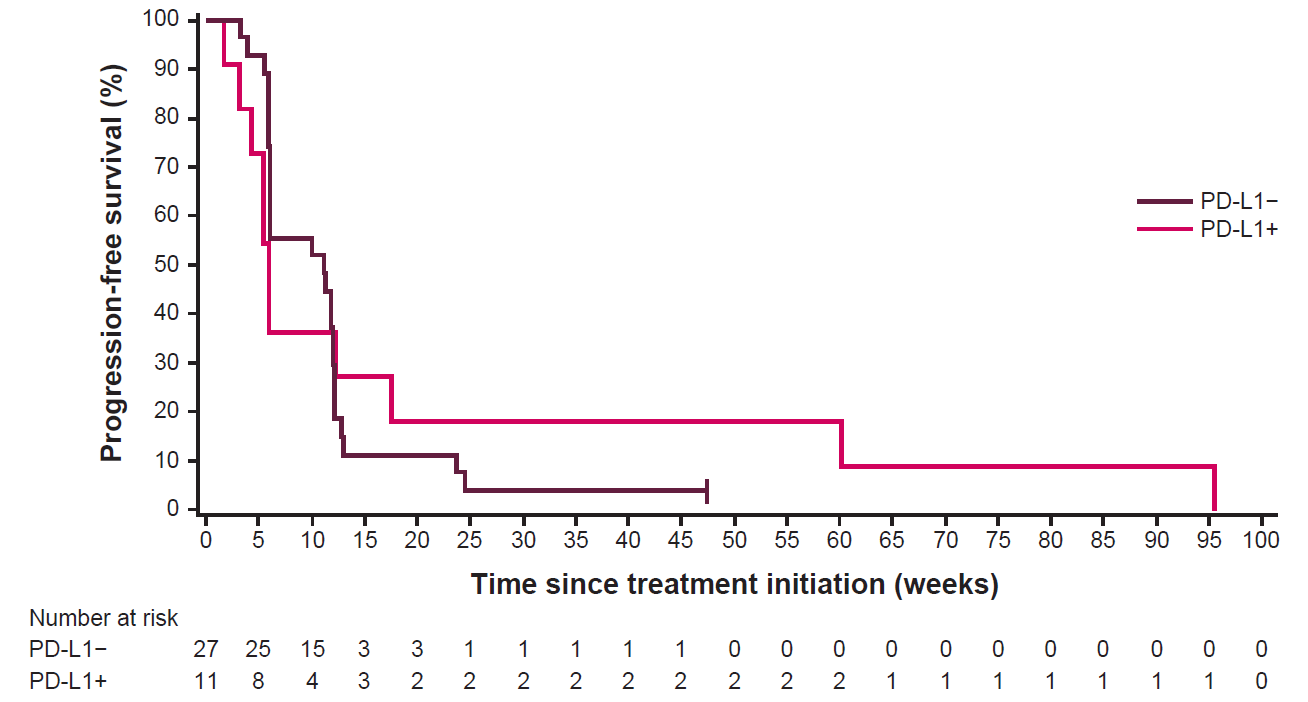


**b**
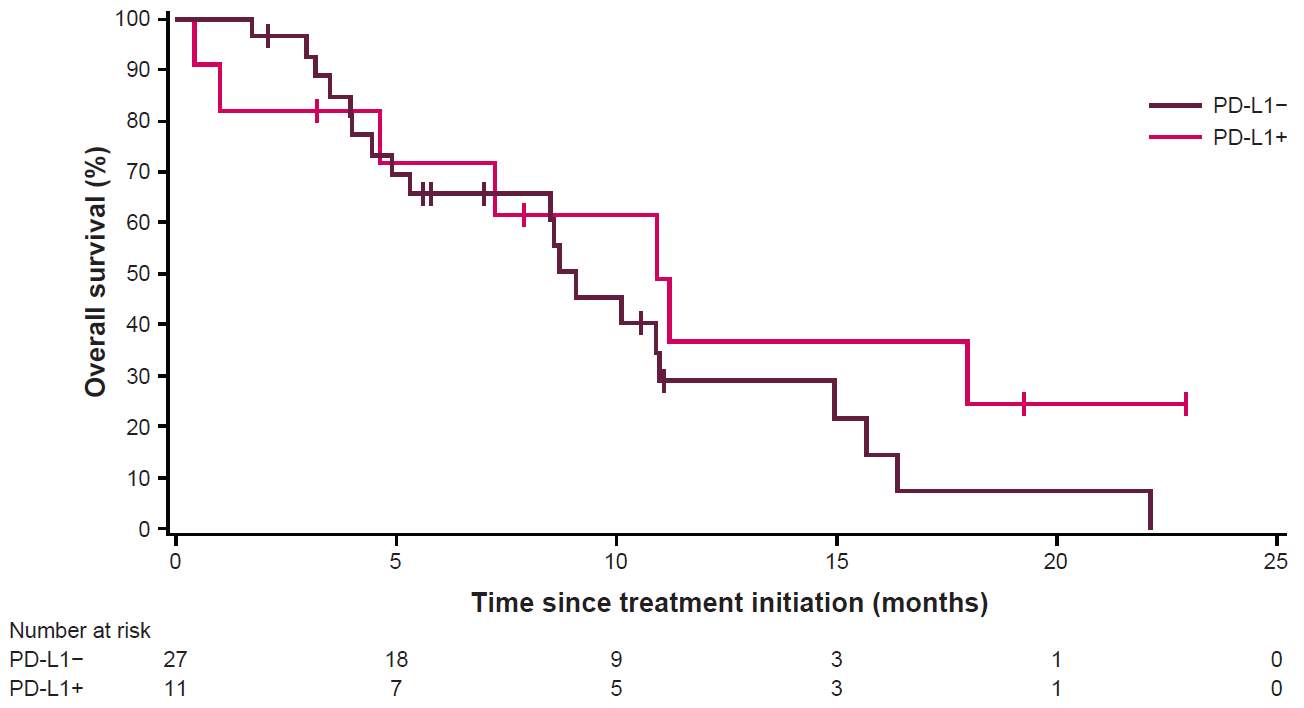

Supplement: Supplementary file 1 — Supplementary material 1 (DOCX 222 KB) [file 10120_2018_903_MOESM1_ESM.docx]
